# Three-Dimensional Choroidal Vessels Assessment in Fellow Eyes of Patients With Central Serous Chorioretinopathy

**DOI:** 10.1167/tvst.14.9.10

**Published:** 2025-09-08

**Authors:** Nicola Valsecchi, Elham Sadeghi, Elli Davis, Mohammed Nasar Ibrahim, Nasiq Hasan, Sandeep Chandra Bollepalli, Sumit Randhir Singh, Luigi Fontana, Jose Alain Sahel, Kiran Kumar Vupparaboina, Jay Chhablani

**Affiliations:** 1 Department of Ophthalmology, University of Pittsburgh, School of Medicine, Pittsburgh, Pennsylvania, USA; 2 Ophthalmology Unit, Dipartimento di Scienze Mediche e Chirurgiche, Alma Mater Studiorum University of Bologna, Bologna, Italy; 3 IRCCS Azienda Ospedaliero-Universitaria di Bologna, Bologna, Italy; 4 Department of Vitreoretina, Akhand Jyoti Eye Hospital, CoE Mastichak, Saran, Bihar, India

**Keywords:** central serous chorioretinopathy, fellow eye, deep learning, three-dimensional, choroid

## Abstract

**Purpose:**

To evaluate choroidal vasculature using a novel three-dimensional algorithm in fellow eyes of patients with unilateral chronic central serous chorioretinopathy (cCSC).

**Methods:**

Patients with unilateral cCSC were retrospectively included. Automated choroidal segmentation was conducted using a deep-learning ResUNet model. Phansalkar thresholding was applied to binarize choroidal vasculature, and three-dimensional maps were created. Mean choroidal vessel diameter, intervessel distance, choroidal thickness, and choroidal vascularity index (CVI) were measured. Linear mixed models were used for statistical analysis.

**Results:**

Thirty unilateral cCSC eyes, 22 fellow, and 26 controls were included. Both cCSC and fellow eyes exhibited significant higher mean choroidal vessel diameter compared with controls (362.50 ± 83.23 µm, 276.84 ± 26.89 µm, and 233.28 ± 28.18 µm, respectively; *P* < 0.001), and in choroidal thickness (288.90 ± 64.77 µm, 269.76 ± 71.17 µm, and 223.97 ± 43.40 µm, respectively; *P* = 0.001). The intervessel distance was reduced in cCSC and fellow eyes compared with controls (196.53 ± 23.58 µm, 225.05 ± 33.72 µm, and 264.13 ± 46.06 µm, respectively; *P* < 0.001). Although lower, the CVI was not significantly different in cCSC and fellow eyes compared with controls (38.14 ± 5.55%, 37.23 ± 6.41%, and 40.65 ± 3.53%, respectively; *P* = 0.066), indicating a possible trend toward a lower CVI.

**Conclusions:**

Three-dimensional representation of choroidal vasculature revealed significant changes in both cCSC and fellow eyes, including a larger diameter and reduced spacing compared with healthy controls.

**Translational Relevance:**

Using a validated deep learning-based three-dimensional method, we observed changes in the choroidal vasculature in both CSC and fellow eyes.

## Introduction

Central serous chorioretinopathy (CSC) is a multifactorial condition within the pachychoroid spectrum disorders, marked by serous retinal detachments, pigment epithelial detachments, and localized or widespread abnormalities of the retinal pigment epithelium (RPE).^1^ It is most commonly observed in young and middle-aged men and is linked to various risk factors, including the use of exogenous systemic corticosteroids, endogenous steroid production, type A personality traits, pregnancy, short axial length, genetic predisposition, and increased scleral thickness.^2^^–^^4^ Abnormalities in choroidal circulation are believed to play a pivotal role in the development of the disease. Hallmarks of CSC include delayed choroidal filling, choroidal vascular dilation, and choroidal vascular hyperpermeability. The disease is thought to result from choroidal congestion owing to impaired vortex vein drainage, leading to choroidal vessel enlargement through dilation and remodeling, increased pressure within the capillaries, and subsequent capillary leakage. This process ultimately damages the RPE and the overlying retina, with severe visual decline.^5^^,^^6^CSC presents a bilateral onset in up to 40% of total cases, and it typically affects one eye initially, with the fellow eye often becoming affected later in life. Previous studies observed abnormalities in the choroid in both the affected and the fellow eye in patients with CSC, including asymmetric venous drainage, choroidal intervortex venous anastomoses, choroidal hyperpermeability, and pachyvessels.^7^ Also, unaffected eyes showed anatomical predisposition to choroidal congestion, including a higher rate of intervortex vein anastomoses at the watershed zones and increased scleral thickness.^8^^–^^13^

Recent advances in optical coherence tomography (OCT) have enhanced imaging capabilities, enabling noninvasive and comprehensive evaluation of the choroid. Through the use of volume scan data and en face images from wide-field OCT, a more detailed analysis of choroidal vascular structures can be achieved. Moreover, deep learning techniques for choroidal vessel segmentation have been introduced, demonstrating significant potential in quantifying choroidal biomarkers.^14^^–^^17^ Using a validated semiautomated algorithm to generate three-dimensional (3D) maps, we detected changes in the choroidal vasculature of patients with age-related macular degeneration, healthy eyes, and eyes affected by diabetic retinopathy.^18^^–^^20^ Given the complex architecture of choroidal vessels, 3D imaging is essential for a more precise and comprehensive evaluation of the choroidal vasculature compared with traditional two-dimensional approaches. Hence, applying this methodology may provide deeper insights into the spatial and anatomical distribution of choroidal vessels in pachychoroid spectrum disorders, including unaffected eyes with unilateral CSC.^18^^,^^19^^,^^21^^,^^22^ Therefore, the main objective of the present study was to assess the choroidal vascular structure with this novel 3D algorithm in affected and fellow eyes of patients with unilateral CSC, and to compare the results with a cohort of age-matched healthy controls.

## Material and Methods

### Study Population

In this retrospective study, we included the eyes of patients with unilateral chronic CSC (cCSC) and healthy age-matched controls. The study was conducted at the Medical Retina and Vitreoretinal Surgery, University of Pittsburgh School of Medicine, from January 2018 to January 2024. The study adhered to the tenets of the Declaration of Helsinki, and informed consent was obtained from all the patients included in the study. We included patients with confirmed diagnosis of unilateral cCSC (onset >3 months), an interval of at least 3 months without treatment, availability of detailed information regarding the clinical history of the patients, and high-definition OCT images.^23^ The inclusion of cCSC patients was aimed at specifically evaluating long-term vascular and structural changes resulting from chronic choroidal remodeling. We excluded patients with a diagnosis of bilateral CSC, ocular diseases such as glaucoma or other optic nerve disorders, advanced cataracts, keratoconus, prior intraocular surgeries (except for uncomplicated cataract surgery), vitreoretinal pathologies, uveitis, diabetic retinopathy, or other vascular disorders in either eye. Additionally, patients with high myopia (>−6 diopters), hyperopia (>+3 diopters), astigmatism (>±3 diopters), or any condition impairing retinal examination or OCT image quality were excluded. Unaffected fellow eyes were defined as eyes without any structural or functional abnormalities related to CSC. Eyes with subtle changes, including pachychoroid pigment epitheliopathy, were excluded to avoid inclusion of subclinical pathology. A cohort of healthy age-matched controls was also included in the study, adhering to the same exclusion criteria.

## OCT Imaging Acquisition and Segmentation

Wide-field swept-source OCT (SS-OCT) scans (12 × 12 mm) were acquired using the Plex Elite 9000 device (Carl Zeiss Meditec, Dublin, CA), centered on the fovea. Only scans with a quality score of 6 or higher, as determined by the in-built scoring system of the SS-OCT software, were included in the analysis. Each OCT volume comprised of 1024 B-scans and the resolution of each scan was 1024 × 1536. Overall, a total of 30 eyes with unilateral CSC and 26 healthy eyes were included. Four unaffected eyes among the unilateral CSC group were excluded owing to poor image quality.

### Automated Choroidal Vessel Segmentation

**Figure 1.**
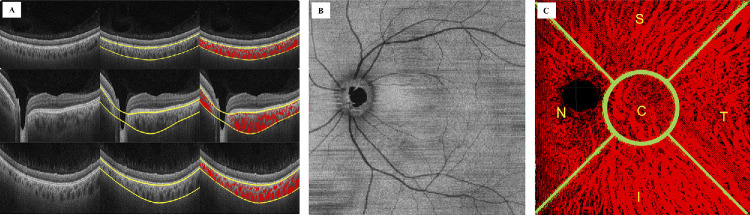
Choroidal vessel segmentation and 3D representation in the fellow eye a patient with unilateral cCSC. (**A**) SS-OCT scans of the left eye of a patient with unilateral cCSC in the right eye. Automated segmentation of the choroidal inner and outer boundary was performed, and choroidal vessels were binarized through the Phansalkar thresholding method. (**B**) En face images are generated to localize the center of the fovea and create the grid. (**C**) The 3D 12  ×  12 mm map is displayed. The grid divides the 3D map in five sectors: nasal (N), temporal (T), superior (S), inferior (I), and central (C). The central sector presents a diameter of 4 mm.

The methodology integrates both automated and manual processes to measure the cross-sectional diameter of 3D choroidal vasculature. First, the boundaries of the choroidal layer were delineated algorithmically, followed by vessel segmentation on individual B-scans. The delineation involved detecting the choroid inner boundary along the choroidal–RPE junction and the choroid outer boundary along the choroidal–scleral junction, as previously described.^18^^–^^20^ Recognizing the effectiveness of deep learning methods for segmenting the choroidal layer, we used our previously established residual encoder–decoder deep learning model, as described in prior studies.^14^^,^^24^ In brief, the inner and outer boundaries of the choroid were identified through a multistep image-processing pipeline involving denoising, contrast enhancement, binarization, morphological filtering, and volumetric smoothing. After reducing speckle noise using median filtering and enhancing contrast with adaptive histogram equalization, initial boundary estimates were obtained via connected component analysis. These estimates were then refined using a combination of robust locally weighted regression and tensor voting to ensure smooth and continuous delineations across the entire 3D volume. Full methodological details can be found in our previously published works.^18^^–^^20^^,^^24^ To address potential inaccuracies in choroid boundary delineation, a manual boundary correction step was integrated into the workflow. In this study, the algorithm achieved an accuracy of 93.33% in delineating the choroidal boundaries in cCSC eyes, 95.45% in fellow eyes, and 92.30% in control eyes, with manual correction required for the remaining cases. Next, we segmented the choroidal vasculature from the SS-OCT volume based on the algorithmically delineated choroidal layer. For this purpose, we used the Phansalkar thresholding method, which has been recently highlighted for its efficacy in choroidal vessel segmentation. This method adaptively estimates local thresholds within overlapping windows of 16 × 16 pixels on each B-scan, effectively differentiating luminal from stromal regions.^25^ After segmentation, postprocessing steps, including morphological operations, were applied to eliminate artifacts and produce a refined 3D choroidal vasculature model. To assist the grader in efficiently performing both automated and manual subtasks within the proposed methodology, we developed two graphical user interfaces (GUIs), GUI-1 and GUI-2. In GUI-1, ImageJ 1.51 s (National Institutes of Health, Bethesda, MD), were used to generate an optic disc mask. This mask was used to exclude the optic disc region from the choroidal vasculature, ensuring accurate 3D choroidal vasculature representation in the OCT volumes. The 3D choroidal vasculature data generated in GUI-1 were transferred to GUI-2 for cross-sectional diameter measurement. In GUI-2, en-face images centered on the fovea were displayed, featuring a grid that divided the 3D vasculature into nasal, temporal, superior, inferior, and central sectors (Fig. 1). The sequence of sectors was displayed on the screen with corresponding labels, guiding the grader through the process. Additionally, a separate window would open, showing the vasculature within a small, fixed-size area around the selected point, assisting the grader in making cross-sectional diameter measurements at the desired locations within that sector. After segmentation of the choroidal vessels and binarization using Phansalkar thresholding, four eyes in the unaffected group were excluded from the analysis owing to insufficient visualization of the choroidal vessels in the 3D maps.

### Assessment of Choroidal Vessel Diameter and Interdistance Between Vessels

The analysis involved selecting the three largest vessels in each sector of the 3D map. Before making the selection, the images were rotated to provide a complete 3D assessment. The vessels were positioned for accurate and thorough evaluation of their diameters. To determine the cross-sectional diameter, measurements were taken from the outermost visible edges of each vessel, with three measurements made for each. These measurements were taken randomly along the straight sections of the vessels, deliberately avoiding areas near bifurcations or the origins of collateral vessels to prevent inaccuracies. In cases of uncertainty, measurements were taken from four or more vessels, with the three largest vessels chosen for analysis to ensure an objective evaluation of the largest vessels in each sector. The mean choroidal vessel diameter (MChVD) for each sector was calculated by averaging the nine measurements (3 per vessel). The intervessel distance (IVD) was defined as the distance between one of the largest vessels and the nearest independent, noncollateral vessel in each sector. To determine the mean IVD, three random measurements were taken for each of the three largest vessels per sector, resulting in a total of nine measurements (3 per vessel). In total, the 15 largest choroidal vessels were measured.^18^^–^^20^ The standard deviation for each individual vessel measurement was evaluated. If the standard deviation exceeded 50 microns for any vessel, the measurement was reassessed and repeated to ensure accuracy. This quality control procedure ensured consistency and reliability across all sectors included in the final analysis. Additionally, the mean value of the five sectors was calculated to provide a global assessment of each choroidal biomarker for each eye. The selection of vessels and measurements was performed by two graders, who were blinded to patient characteristics (N.V. and E.D.). The measurements from the first grader (N.V.) were used for analysis, and the measurements from the second grader (E.D.) were used to assess intergrader reliability. Using the ResUnet model for segmentation and the Phansalkar thresholding method for binarization, we determined the choroidal thickness and choroidal vascularity index (CVI) across the entire volume.

### Statistical Analysis

Normality was assessed using the Shapiro-Wilk test, and parametric tests were applied accordingly (Supplementary Table 1). The inter-rater reliability for vessel measurements obtained from image binarization was evaluated using the absolute agreement model of the intraclass correlation coefficient (ICC). ICC values were interpreted as follows: less than 0.50 indicated poor reliability, between 0.50 and 0.75 indicated moderate reliability, between 0.75 and 0.90 indicated good reliability, and greater than 0.90 indicated excellent reliability. The Fisher test was used for analyzing categorical variables. Linear mixed models were used to compare demographic data, CTh, CVI, MChVD, and IVD between unilateral cCSC, fellow, and healthy eyes. Random intercepts were included to account for within-subject variability; random slopes were not specified. The Bonferroni correction was applied for multiple comparisons. Pearson correlation was used to assess the correlation between the mean choroidal parameters. A *P* value of less than 0.05 was considered statistically significant. Statistical analysis was performed using IBM Statistical Package for the Social Sciences (SPSS) Statistics version 26 (IBM, Armonk, NY).

## Results

### Demographic Data

A total of 30 eyes of 30 patients with unilateral cCSC, 22 fellow eyes, and 26 eyes of 26 healthy individuals were included in the study. No differences in age and sex were observed between the three groups (*P* > 0.05). Visual acuity was significantly reduced in cCSC group compared with fellow eyes and controls (*P* < 0.001). In contrast, no differences in the rate of pseudophakia were observed between the three groups (*P* = 0.141) (Table 1). Among the cCSC group, the mean time from the onset of the disease was 58.52 ± 16.35 months. Nine eyes (30%) were treatment naïve, 15 eyes (50%) received previous photodynamic therapy (PDT), 4 (13.33%) eyes received anti-vascular endothelial growth factor (VEGF) treatment for associated choroidal neovascularization, and 2 eyes (6.67%) received both PDT and anti-VEGF treatment.

**Table 1.** Demographic Data Among the Three Different Groups

	cCSC Eyes (*n* = 30)	Fellow Eyes (*n* = 22)	Healthy Eyes (*n* = 26)	*P* Value
Age, years	54.63 ± 11.69	53.63 ± 12.95	50.64 ± 23.17	0.665
Female sex	8 (26.67%)	6 (27.27%)	14 (53.84%)	0.065
BCVA, logMAR	0.24 ± 0.20	0.02 ± 0.04	0.01 ± 0.03	**0.001**
Pseudophakia	2 (6.67%)	3 (13.63%)	4 (15.38%)	0.141

BCVA, best-corrected visual acuity; logMAR, logarithm of the minimum angle of resolution.

Values are mean ± standard deviation or number (%).

### Choroidal Biomarkers in Different Groups

Overall, the measurements by both readers showed a good level of agreement (ICC = 0.877; 95% confidence interval [CI], 0.811–0.914). The mean CTh was significantly higher in cCSC and fellow eyes compared with controls (*P* < 0.001). After Bonferroni correction, the difference between cCSC and healthy eyes (*P* < 0.001), and fellow and healthy eyes (*P* = 0.033) remained statistically significant. In contrast, the difference was not statistically significant when cCSC eyes were compared with fellow eyes (*P* = 0.792). The central sector presented the highest CTh, whereas the nasal sector presented the lowest values of CTh for each group. The mean CVI was reduced in cCSC and fellow eyes compared with healthy controls, but the difference did not attain statistical significance (*P* = 0.066). The only sector with a significant difference in the CVI was the nasal **(**Table 2).

**Table 2.** Choroidal Thickness and CVI Among the Three Different Groups

	cCSC Eyes (*n* = 30)	Fellow Eyes (*n* = 22)	Healthy Eyes (*n* = 26)	*P* Value
Choroidal thickness, µm				
Mean	288. 90 ± 64.77	269.76 ± 71.17	223.97 ± 43.40	**0.001**
Nasal	251.49 ± 62.73	237.42 ± 71.66	204.87 ± 62.18	**0.030**
Temporal	276.94 ±63.30	271.27 ± 77. 97	232.52 ± 48.28	**0.044**
Inferior	289.88 ± 77.33	244.83 ± 63.11	235.18 ± 39.29	**0.004**
Superior	271.54 ± 71.68	267.13 ± 65.51	205. 91 ± 45.49	**<0.001**
Central	364.62 ± 77.33	338.94 ± 91.12	251.03 ± 54.40	**<0.001**
CVI, %				
Mean	38.14 ± 5.55	37.23 ± 6.41	40.65 ± 3.53	0.066
Nasal	38.83 ± 3.64	37.33 ± 3.00	40.58 ± 5.35	**0.033**
Temporal	39.31 ± 2.22	38.16 ± 2.98	40.35 ± 4.28	0.097
Inferior	40.63 ± 3.50	40.25 ± 3.79	42.15 ± 3.54	0.154
Superior	41.81 ± 2.30	41.27 ± 2.74	41.12 ± 3.63	0.667
Central	39.53 ± 2.47	39.68 ± 2.39	40.64 ± 3.51	0.318

Values are mean ± standard deviation. Significant values are in bold.

**Figure 2.**
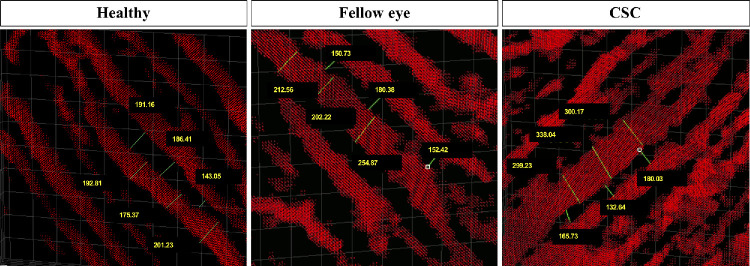
Choroidal vessels in the inferior sectors in a healthy eye, an eye affected by unilateral CSC, and the fellow eye. The image on the *left* shows the choroidal vessels in the inferior sector in the left eye of a healthy 36-year-old man. The image on the center shows the fellow eye of a 38-year-old man affected by CSC in the right eye. The image on the *right* shows the right eye of the same patient. Note how the choroidal vessels are engorged and the IVD reduced in CSC and fellow eyes compared with the healthy eye (measurements shown are in micrometers).

**Figure 3.**
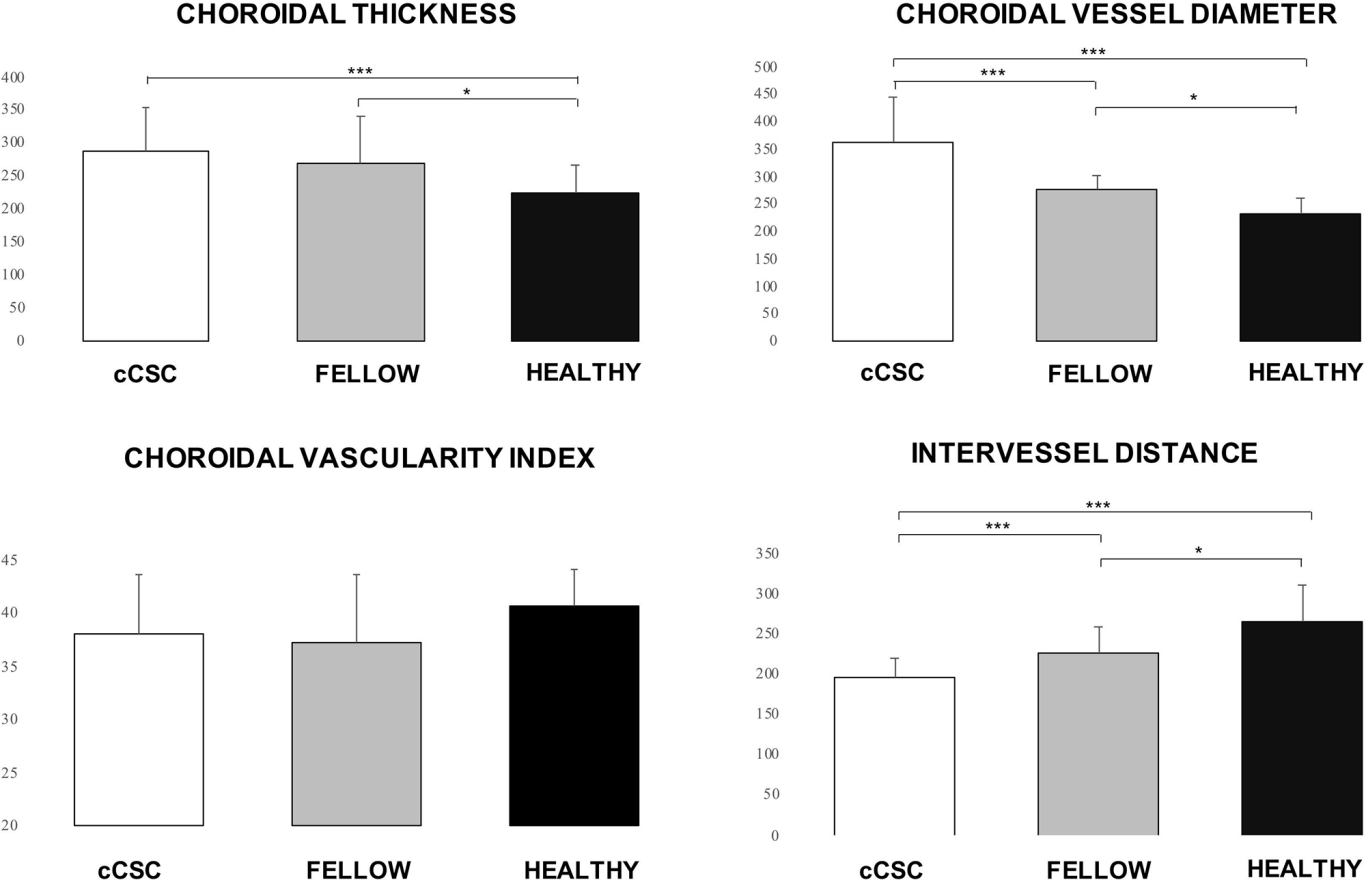
Graphical representation of mean choroidal parameters across distinct study groups. On the vertical axis, measurements for choroidal thickness (ChT), MChVD, and IVD are reported in micrometers (µm), and values for CVI are expressed as percentages.

The mean MChVD was significantly higher in cCSC and fellow eyes compared with controls (*P* < 0.001). After Bonferroni correction, the difference between cCSC and healthy eyes (*P* < 0.001), and fellow and healthy eyes (*P* = 0.033) remained statistically significant. Furthermore, cCSC eyes presented a higher mean MChVD compared with fellow eyes (*P* < 0.001). The sector with the highest MChVD was the inferior, whereas the sector with the lowest MChVD was the nasal for each group. The mean IVD was significantly lower in cCSC and fellow eyes compared with controls (*P* < 0.001). After Bonferroni correction, the difference between cCSC and healthy eyes (*P* < 0.001), and fellow and healthy eyes (*P* = 0.001) remained statistically significant. Also, cCSC eyes presented a significant reduction in IVD compared with fellow eyes (*P* = 0.015) (Table 3). A representative example is shown in Figures 2 and 3.

**Table 3.** MChVD and IVD Among the Three Different Groups

	cCSC Eyes (*n* = 30)	Fellow Eyes (*n* = 22)	Healthy Eyes (*n* = 26)	*P* Value
MChVD, µm				
Mean	362.50 ± 83.23	276.84 ± 26.89	233.28 ± 28.18	**<0.001**
Nasal	341.57 ± 84.19	263.32 ± 44.19	220.12 ± 35.68	**<0.001**
Temporal	365.82 ± 98.78	281.72 ± 43.39	239. 93 ± 38.35	**<0.001**
Inferior	376.89 ± 91.04	289.85 ± 38.86	250.45 ± 41.12	**<0.001**
Superior	370.99 ± 289.94	289.14 ± 47.61	239.62 ± 43.83	**<0.001**
Central	357.26 ± 84.50	261.05 ± 43.30	224. 93 ± 48.52	**<0.001**
IVD, µm				
Mean	196.53 ± 23.58	225.05 ± 33.72	264.13 ± 46.06	**<0.001**
Nasal	189.70 ± 34.86	219.20 ± 42.89	255.60 ± 70.06	**<0.001**
Temporal	194.30 ± 44.57	235.37 ± 51.96	278.41 ± 59.44	**<0.001**
Inferior	192.96 ± 36. 95	229.41 ± 60.82	238.76 ± 68.04	**0.007**
Superior	202.66 ± 39.95	221.23 ± 58.98	259.31 ± 71.06	**0.002**
Central	203.02 ± 41.90	200.04 ± 41.90	300.13 ± 76.68	**<0.001**

Values are mean ± standard deviation. Significant values are in bold.

Overall, we observed that the mean MChVD was correlated with the mean CTh (r = 0.547; *P* < 0.001). In contrast, we did not observe any correlations between mean CVI and MChVD (r = 0.168; *P* = 0.375), mean CVI and CTh (r = 0.384; *P* = 0.055), mean CVI and IVD (r = −0.096; *P* = 0.615), mean IVD and MChVD (r = −0.187; *P* = 0.322), or mean IVD and CTh (r = 0.120; *P* = 0.526). We found a significant correlation between mean CTh and mean MChVD in cCSC and fellow eye groups (r = 0.562; *P* < 0.001; and r = 0.499; *P* = 0.018, respectively), but no significant correlation in the healthy group (r = −0.028; *P* = 0.894). When we compared treatment naïve cCSC and previously treated patients, we did not observe any significant differences among the two groups (*P* > 0.05) (Supplementary Table 2).

## Discussion

Using a deep learning-based 3D method to evaluate choroidal vessels, we found that both cCSC eyes and their fellow eyes had a significantly higher mean MChVD and CTh compared with healthy eyes. Additionally, the mean IVD was significantly reduced in cCSC and fellow eyes compared with controls. Although the CVI was also reduced in cCSC and fellow eyes compared with controls, the difference was not statistically significant, indicating a possible trend toward a lower CVI. We also observed a significant correlation between CTh and mean MChVD in the cCSC group and in the fellow eyes. However, no significant correlation was found in the healthy group.

In this study, we used a novel semiautomated deep-learning algorithm to generate 3D visualizations of choroidal vessels using SS-OCT volumes, as previously described.^18^^–^^20^ This approach enabled a comprehensive noninvasive analysis of the choroidal vasculature. Previous research has predominantly relied on OCT B-scans or volumetric reconstructions to evaluate choroidal biomarkers. Xiao et al.^26^ observed increased values of CVI, CT, choroidal vessel volume and choroidal stromal volume in treatment-naïve acute CSC and fellow eyes compared with controls. Also, Zeng et al.^27^ observed an increased CVI in CSC eyes compared with controls in ultra-field SS-OCTA scans. Furthermore, Hara et al.^28^ reported decreased choroidal vessel volume in patients with CSC in response to PDT and laser photocoagulation, especially in the treated area. However, these methods are limited in their ability to comprehensively assess the choroidal vasculature, particularly in measuring individual vessel diameters and understanding the relationships between vessels, capabilities that are uniquely achievable through 3D imaging techniques.^19^ Hence, the use of 3D maps provides a comprehensive assessment of the relationships between vessels, enhancing our understanding of the anatomical connections within the choroid.

Previous studies have reported choroidal engorgement in CSC, including in both the affected and fellow eyes, as well as findings such as hyperpermeability, pachyvessels, and intervortex vein anastomoses. However, no prior study has used a 3D approach to evaluate the choroidal vasculature in different sectors of CSC and fellow eyes. Our results showed that cCSC and fellow eyes displayed a higher CTh and MChVD compared with healthy controls. These findings support the involvement of choroidal congestion in the pathogenesis of CSC. Moreover, we found a correlation between CTh and MChVD in CSC and fellow eyes, a relationship not observed in healthy controls.

Another finding in this study was the lower mean IVD in both cCSC and fellow eyes compared with healthy controls. The IVD is a novel biomarker that measures the 3D relationships among choroidal vessels in Haller's layer. In conditions characterized by choroidal congestion, the decrease in IVD might be explained by a decrease in the space between vessels within this layer. Matsumoto et al.^29^ described the presence of venous anastomoses within the vortex vein system as a mechanism to relieve venous congestion in pachychoroid spectrum disorders. Also, several studies have noted that these anastomoses are more common in both affected and unaffected eyes compared with healthy controls.^7^^,^^8^ Brinks et al.^30^ suggested that abnormal arteriovenous connections could contribute to the progression of cCSC. These arteriovenous anastomoses may bypass the normal choriocapillaris route, directly filling veins with arterial blood and leading to choroidal venous congestion.^30^ Therefore, another possible explanation for the reduced IVD in cCSC and fellow eyes observed in the present study could be secondary to the presence of arteriovenous and venous-venous anastomoses in both the macula and peripheral retina, occurring in the context of choroidal congestion.

Overall, our results indicate that fellow eyes, although clinically unaffected, exhibit choroidal vascular architecture closely resembling that of CSC eyes. This similarity, characterized by increased choroidal thickness and vessel diameter compared with healthy controls, may reflect an underlying predisposition to disease development, likely driven by subclinical choroidal congestion and vascular remodeling. Additionally, the lower IVD observed in fellow eyes may represent early vascular changes that disrupt the normal spatial arrangement of choroidal vessels. Such alterations could impair hemodynamics, which in the context of choroidal engorgement may compromise choriocapillaris perfusion and increase mechanical stress on the RPE. These pathophysiological processes could predispose fellow eyes to develop CSC over time, even in the absence of clinical symptoms at baseline. Therefore, future longitudinal studies combining 3D vascular imaging with indocyanine green angiography mapping could provide further insights into the mechanisms of vascular remodeling and disease evolution in fellow eyes.

Previous studies have shown a higher CVI in acute cCSC compared with healthy individuals, suggesting its potential as a reliable biomarker for acute cases. However, findings for CVI in chronic cCSC have been inconsistent. Recently, Kaye et al.^31^ reported a lower CVI in chronic cCSC and fellow eyes compared with controls when assessing the central subfoveal choroidal area. In contrast, Sahoo et al.^32^ observed a trend of a higher CVI toward the center of the macula and superiorly in both acute and cCSC eyes compared with controls using 6 × 6 mm scans, although the difference was not statistically significant. Additionally, Zeng et al.^27^ found an increased CVI in acute and cCSC and fellow eyes compared with controls in 20 × 24 scans. These conflicting results likely arise from various factors, including the method used to quantify CVI, the extension of the area assessed, and the stage at which CSC eyes are evaluated. Although we used a novel 3D CVI analysis over a wide 12 × 12 mm area, our study did not reveal statistically significant differences in CVI between cCSC, fellow, and control eyes, despite a trend toward lower values in cCSC and fellow eyes. These findings suggest that CVI may have limited utility as a biomarker in cCSC.

Furthermore, we found that both CTh and MChVD were highest in the inferior sectors across all groups and lowest in the nasal sectors. Several studies have reported lower choroidal thickness in the nasal sector, possibly owing to the nasal positioning of the optic disc influencing choroidal distribution. However, findings regarding the thickest choroidal sectors have been inconsistent, likely owing to variations in methodology and the size of the analyzed maps.^33^^–^^35^ In a previous study assessing 6 × 6 mm choroidal maps in healthy patients using the same validated 3D method, we observed that the thickest region was central, while the sector with the highest MChVD was inferior.^19^ In contrast, the higher MChVD in the inferior sector observed in the present study using 12 × 12 mm maps may be attributed to gravity-dependent blood flow, which enhances circulation in that area and is more effectively captured with wider-field scans.

In the present study, we did not observe any significant differences between treatment-naïve and previously treated cCSC eyes. Conflicting results have been reported regarding the effects of PDT and anti-VEGF treatment on choroidal biomarkers in cCSC. Bui et al.^36^ observed a decrease in CVI and SFCT 12 months after half-fluence PDT. Similarly, Chung et al.^37^ reported a decrease in choroidal thickness following anti-VEGF therapy. In contrast, Van Rijssen et al.^38^ found no significant changes in CVI after PDT, suggesting that treatment effects may be more closely related to changes in vessel wall structure and permeability rather than to luminal density. In the present study, the small sample size and heterogeneity of the study groups may have impacted the reliability of our findings. Consequently, larger and more homogeneous cohorts are needed in future studies to better elucidate the potential influence of different treatment modalities on 3D choroidal biomarkers.

The limitations of our study include its retrospective design and small sample size. In particular, the small sample size of fellow eyes resulted from predefined exclusions owing to inadequate image quality and segmentation errors. Although this factor may reduce statistical power and generalizability, all included data met rigorous quality control criteria. Our future objective is to improve software analysis algorithms to minimize the exclusion of images owing to inadequate quality and segmentation. Additionally, our analysis focused on a 12 × 12 mm area, and we did not assess the peripheral choroid or the vortex veins. To validate our findings, larger prospective studies with a broader patient cohort are needed. Also, this study did not include axial length measurements for all participants, which could influence choroidal thickness and vessel parameters. Although we excluded patients with significant refractive errors, future work will incorporate axial length data to better address its potential confounding effects. Another limitation is that the process of selecting and measuring the three largest choroidal vessels and IVD per sector required evaluation by trained observers. To ensure objective measurements, images were rotated to offer a comprehensive 3D view, and only the three largest vessels in each sector were included in the analysis. We found good intergrader reliability, suggesting consistency in the measurements. However, further research is required to fully automate the process of selecting the largest vessels, measuring IVDs per sector, and performing these measurements automatically. Furthermore, we aim to investigate the spatial relationship between PDT application zones and choroidal vascular alterations using widefield OCT angiography to better characterize the localized and global effects of PDT on choroidal remodeling in cCSC. Also, our next objective is to conduct longitudinal studies to determine whether specific vascular features could serve as predictive biomarkers for the development of CSC in fellow eyes.

## Conclusions

We observed a higher in CTh and MChVD in both the affected eyes with unilateral cCSC and their fellow eyes compared with controls. Additionally, there was a decrease in the mean IVD in cCSC and fellow eyes compared with controls. Overall, these results suggest that the choroidal vasculature is affected in fellow eyes of patients with unilateral cCSC, with a larger diameter of the vessels and a reduced spacing. The use of a deep-learning approach for 3D evaluation of choroidal vessels is an innovative, noninvasive technique that facilitates a detailed analysis of vessel morphology, diameters, and spatial location. Therefore, this technology holds great potential for deepening our understanding of the pathophysiological mechanisms underlying CSC pathogenesis, with the prospect of future clinical applications.

## Supplementary Material

Supplement 1

Supplement 2
