# Supplementary material for: Three-Dimensional Choroidal Vessels Assessment in Fellow Eyes of Patients With Central Serous Chorioretinopathy
Source: Transl Vis Sci Technol. 2025 Sep 8;14(9):10. doi: 10.1167/tvst.14.9.10 (PMC12422394; doi:10.1167/tvst.14.9.10)
Supplement: Supplement 1 [file tvst-14-9-10_s001.pdf]

Variables	Shapiro-Wilk test	
	Statistic	p-value
**Age**	0.969	0.145
**BCVA**	0.538	0.057
**Mean MChVD**	0.908	0.054
**Mean IVD**	0. 972	0.205
**Mean CT**	0.955	0.067
**Mean CVI**	0.968	0.132
**Nasal MChVD**	0.944	0.068
**Nasal IVD**	0.846	0.052
**Nasal CT**	0.955	0.071
**Nasal CVI**	0.950	0.053
**Temporal MChVD**	0.909	0.052
**Temporal IVD**	0. 971	0.177
**Temporal CT**	0.947	0.063
**Temporal CVI**	0.978	0.373
**Superior MChVD**	0.908	0.056
**Superior IVD**	0.902	0.055
**Superior CT**	0.978	0.374
**Superior CVI**	0.975	0.272
**Inferior MChVD**	0.954	0.057
**Inferior IVD**	0.957	0.058
**Inferior CT**	0.955	0.061
**Inferior CVI**	0.988	0.836
**Central MChVD**	0.951	0.053
**Central IVD**	0.988	0.056
**Central CT**	0.965	0.089
**Central CVI**	0.954	0.068

Supplementary table 1. The Shapiro-Wilk test is shown in the table. BCVA=best corrected visual acuity, MChVD=mean choroidal vessel diameter, IVD=inter-vessel distance, CT=choroidal thickness, CVI=choroidal vascularity index.
