# Supplementary material for: Three-Dimensional Choroidal Vessels Assessment in Fellow Eyes of Patients With Central Serous Chorioretinopathy
Source: Transl Vis Sci Technol. 2025 Sep 8;14(9):10. doi: 10.1167/tvst.14.9.10 (PMC12422394; doi:10.1167/tvst.14.9.10)
Supplement: Supplement 2 [file tvst-14-9-10_s002.pdf]

	Treatment-naïve cCSC eyes (n=9)	Previously treated cCSC eyes (n=21)	P value
**Choroidal thickness (μm), mean ± SD**			
**Mean**	315.10 ± 66.58	277.67 ± 62.19	0.150
**Nasal**	267.73 ±69.19	244.54 ± 60. 17	0.363
**Temporal**	298.59 ± 59.02	253.38 ± 61.43	0.072
**Inferior**	334.85 ± 68.38	280.60 ± 74.17	0.055
**Superior**	280.71 ± 75.45	267.61 ± 71.55	0.654
**Central**	393.60 ± 73.78	352.20 ± 77.17	0.184
**Choroidal vascularity index, %**			
**Mean**	39.96 ± 3.52	37.36 ± .6.73	0.251
**Nasal**	39.56 ± 2.18	38.52 ± 4.11	0.486
**Temporal**	39.65 ± 2.10	38.67 ± 2.44	0.294
**Inferior**	40.67 ± 2.23	40.62 ± 3.98	0.974
**Superior**	41.44 ± 2.24	42 ± 2.37	0.565
**Central**	39.44 ± 2.55	39.57 ± 2.50	0.900
**Mean choroidal vessel diameter (MChVD) (μm), mean ± SD**			
**Mean**	415.43 ± 46.46	339.82 ± 85.91	0.055
**Nasal**	403.45 ± 49.85	335.05 ± 82.59	0.056
**Temporal**	404.19 ± 74.35	349.37 ± 104.84	0.168
**Inferior**	449.38 ± 73.74	355.82 ± 111.02	0.063
**Superior**	432.29 ± 73.95	364.72 ± 80.52	0.061
**Central**	387.86 ± 61.75	344.14 ± 90.69	0.199
**Inter-vessel distance (IVD) (μm), mean ± SD**			
**Mean**	194.86 ± 27.01	197.25 ± 22.64	0.804
**Nasal**	169.98 ± 31.67	198.16 ± 33.34	0.051
**Temporal**	199 ± 48.62	192.29 ± 43.83	0.713
**Inferior**	176.97 ± 31.78	199.81 ± 37.57	0.123
**Superior**	217.13 ± 36.80	196.46 ± 40.47	0.199
**Central**	211.22 ± 31.50	199.50 ± 30.11	0.343

Supplementary table 2. Comparison of choroidal biomarkers between treatment-naïve and previously treated eyes with cCSC. cCSC= chronic central serous chorioretinopathy. SD=standard deviation. Significant values are in bold.
